# Gene Polymorphisms and Pharmacogenetics in Rheumatoid Arthritis

**DOI:** 10.2174/138920208785699553

**Published:** 2008-09

**Authors:** Ignacio Rego-Pérez, Mercedes Fernández-Moreno, Francisco J Blanco

**Affiliations:** Osteoarticular and Aging Research Lab, Genomic Unit, Rheumatology Division, Juan Canalejo Hospital, Xubias 84 15006- A Coruña, Spain

**Keywords:** Pharmacogenomic, rheumatoid arthritis, TNF, IL-1, cytokines, gene polymorphisms.

## Abstract

Rheumatoid arthritis (RA) is a systemic, chronic and inflammatory disease of unknown etiology with genetic predisposition. The advent of new biological agents, as well as the more traditional disease-modifying antirheumatic drugs, has resulted in highly efficient therapies for reducing the symptoms and signs of RA; however, not all patients show the same level of response in disease progression to these therapies. These variations suggest that RA patients may have different genetic regulatory mechanisms. The extensive polymorphisms revealed in non-coding gene-regulatory regions in the immune system, as well as genetic variations in drug-metabolizing enzymes, suggest that this type of variation is of functional and evolutionary importance and may provide clues for developing new therapeutic strategies. Pharmacogenetics is a rapidly advancing area of research that holds the promise that therapies will soon be tailored to an individual patient’s genetic profile.

## INTRODUCTION

Rheumatoid arthritis (RA) is a chronic, systemic and inflammatory joint disease that leads to bone and cartilage destruction, as well as a wide variety of extra-articular manifestations. The hyperplastic synovial membrane mediates this process by invading deeply into articular cartilage and bone. During this process, a variety of both inflammatory and noninflammatory mediators, including proinflammatory cytokines, such as interleukin-1β (IL-1β) and tumor necrosis factor-α (TNF-α), the metalloproteinases (MMPs), CD4+ cells, B lymphocytes, macrophages and synovial fibroblasts, contribute to the pathogenesis of RA.

Although well proven, the efficacy of the new drugs used to treat RA is variable. While there are no useful and reliable clinical or molecular markers for response to therapy, the levels of various cytokines and other mediators of inflammation may correlate with the efficacy of the therapies. The main focus of pharmacogenetics up to the present has been on the study of drug-metabolizing enzymes but polymorphisms in drug targets as well as transporters are now also under investigation [1]. 

In this review, we report the clinical influence of some of the gene polymorphisms associated with RA and the principles of pharmacogenetics applied to different therapies, such as classical disease-modifying antirheumatic drugs (DMARDs) and the new biological agents. In the near future, pharmacogenetic studies may make it possible to precisely select medications and dosages for individual patients [1]. 

## CLINICAL INFLUENCE OF GENE POLYMORPHISMS IN RA

Many diseases are multifactorial; both environmental and genetic factors contribute to etiology and/or clinical severity. The genetics of many multifactorial diseases is complex, involving multiple genes. Mendelian patterns of inheritance often do not apply. The genetic contribution to susceptibility for RA is demonstrated by family clustering and a three-fold to four-fold higher pair-wise concordance percentage for clinically expressed RA in monozygotic twins compared to dizygotic twins [2]. Estimates from several authors suggest that at least 10 different genetic regions may be related to RA [3]. The variability in the contribution of the multiple genetic factors involved in RA may be related to the variability of severity seen in clinical presentation. The variability in response to drugs is similarly greater across the population than within the same patient or between monozygotic twins. Part of this difference is attributable to genetic factors [4].

Most of the genes implicated in predisposition to RA are within the HLA-DR loci [5], however, these loci are not the only contributors. Other attractive candidates include the genes for cytokines. Cytokines are important mediators of the inflammatory response and play an important role in the pathophysiology of joint inflammation and destruction in RA [6].

### The HLA Complex

One genetic region consistently shown to be associated with RA is the Major Histocompatibility Complex (MHC). The contribution from this region is estimated to be as much as 30% of the total genetic effect [7]. RA is associated with specific HLA-DRB1 alleles that encode a conserved sequence of amino acids (residues 70-74 in the DRβ1 chain) known as the shared epitope (SE) [8]. This sequence is in the peptide-binding groove. The alleles carrying this sequence are DRB1*0401, DRB1*0404, DRB1*0405, DRB1*0408, DRB1*0101, DRB1*0102 and DRB1*1001 [9]. The presence and gene dosage of HLA-DRB1 alleles encoding the SE have been associated with the presence of rheumatic nodules, a more rapid rate of development of erosions seen in radiography, vasculitis, Felty´s syndrome, and increased need for joint surgery [10]. Interestingly, the DRB1*0401/ *0404 genotype appears to be particularly associated with early disease onset and a more severe disease phenotype [9].

DNA microsatellites in the HLA region have been described. The HLA-B-associated transcript 2 (BAT2) and D6S273 are HLA class III microsatellites, whereas D6S2223 is a HLA class I microsatellite marker [11]. In this review we´ll describe how some of these microsatellite markers have been shown to be associated with the response to therapies.

### Cytokine Genes in RA

Considering the critical role that several cytokines, such as TNF and IL-1, are thought to play in the pathogenesis of RA, the genetic variation of these cytokines and their actual presence in the joint, it is possible that polymorphisms regulating cytokine production affect the natural course of the disease [12]. A number of polymorphisms with possible functional phenotypes have recently been identified, most often in the promoter regions for several of the cytokines (Table **1**); these are thought to be important in maintaining the balance between proinflammatory and anti-inflammatory cytokines.

#### 1. TNF

One of the molecules shown to play a key role in pathogenesis of RA is the proinflammatory cytokine TNF. This cytokine belongs to a family of proteins involved in immune regulation and programmed cell death. In patients with RA, TNF levels are chronically elevated in the blood and, more specifically, in the joints [13]. The functions of TNF are mediated by two distinct TNF receptors, TNFRSF1A and TNFRSF1B, which exist both as monomers on cell surfaces and in soluble forms [14]. TNF has been shown to be involved in the stimulation of cytokine production, enhancing the expression of adhesion molecules, and in neutrophil activation. TNF is also a co-stimulator of T-cell activation and antibody production by B cells [15]. TNF, therefore, contributes to the regulation of normal homeostasis as well as playing an important role in inflammation. Approximately 60% of the variation in TNF production may be genetically determined, indicating a strong genetic influence on the production of cytokines [16]. These observations, in conjunction with the localization of the TNF gene on chromosome 6 within the MHC class III region between the HLA-B and HLA-DR genes [17], prompted speculation about the existence of functionally-relevant polymorphisms in the TNF gene. As a consequence, the TNF gene has received considerable attention as a candidate disease-associated gene in RA. 

**Fig. (1):**
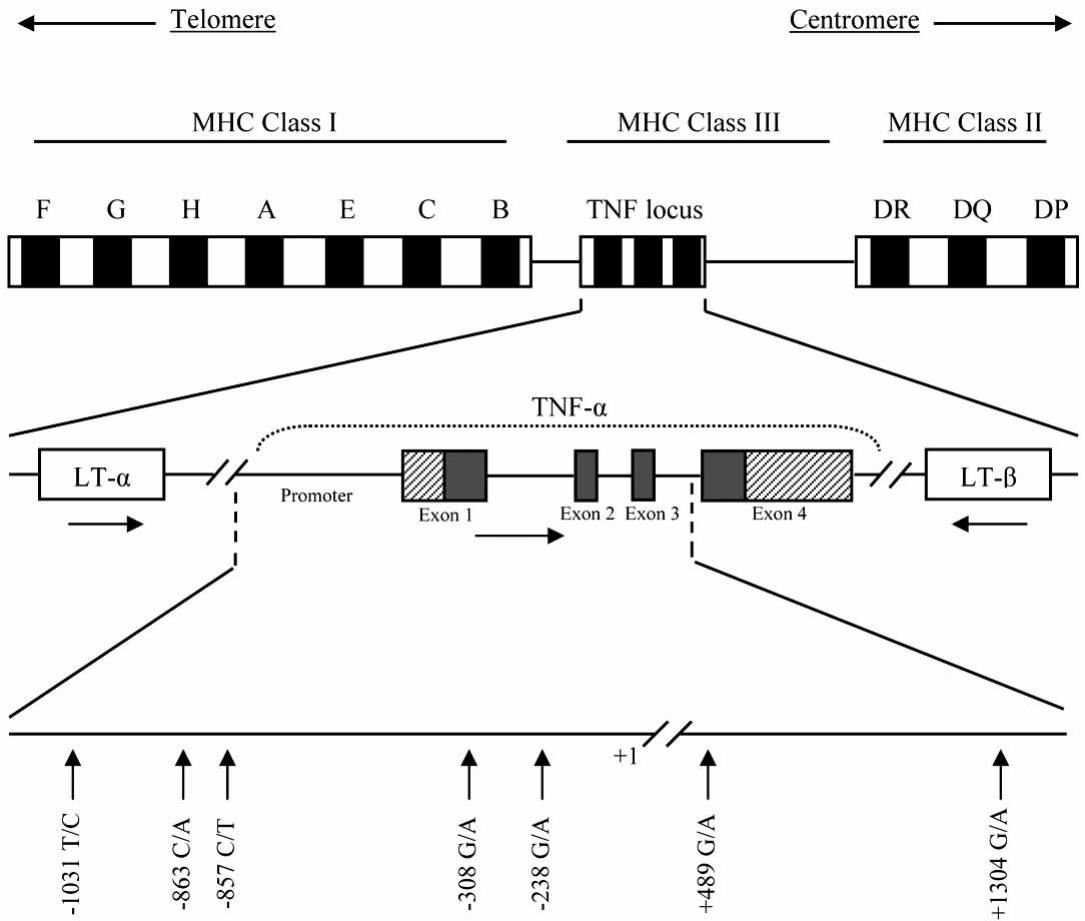
Schematic representation of the tumor necrosis factor-α (TNF-α) gene, showing some of the most relevant single nucleotide polymorphisms (SNPs). The horizontal arrows in the middle row indicate the transcriptional orientation of the TNF and lymphotoxin (LT) genes. Diagonally highlighted regions in exons 1 and 4 indicate the untranslated regions (UTRs).

Within the TNF gene, most of the single nucleotide polymorphisms (SNPs) have been located to the promoter region (Fig. **1**). The first polymorphism identified is a guanine (G) to adenine (A) transition at position -308. The uncommon A allele is strongly associated with the HLA-A1-B8-DR3-DQ2 haplotype [18], and is also associated with both autoimmune diseases and the high TNF producer phenotype [19]. This allele could facilitate deregulation of the cytokine network, affecting the pathology of RA [16]. 

Studies of polymorphism at position -238 showed either a G or the less common A allele at this site. Of the three possible genotypes on the two chromosomes, GG and GA are the most common. The GG genotype appears to be associated with more severe articular erosions; the cartilage of those patients with the GA genotype deteriorated more slowly [20]. Studies have shown a similar association with the site +489, where the GG genotype is also associated with more severe erosive disease [16]. 

These polymorphisms, as well as others within this gene, such as -1031 T/C, -863 C/A, -857 C/T or +1304 G/A, may contribute to susceptibility to RA by increasing TNF-α production [21-24]. They take part in several haplotypes because of the large number of potentially relevant polymorphisms and the complex patterns of linkage disequilibrium that occurs in the MHC region [25].

A SNP has also been described in exon 6 of the TNF-α receptor type 2 (TNFRSF1B). This polymorphism consists of a single base substitution at codon 196 (A**T**G to A**G**G) leading to a nonconservative amino acid change (methionine to arginine). The 196G allele may increase IL-6 production more than the 196T allele. The 196G allele may also affect membrane receptor shedding and/or ligand binding [26].

DNA microsatellites in the TNF locus have also been described. These repeated sequences of bases A and T found in the non-coding regions of DNA serve as genetic markers when they occur in linkage disequilibrium with a nearby functional polymorphism [27]. The TNF locus has five microsatellites (TNFa through TNFe) based on the number of repeated sequences [28]. *In vitro* studies suggest that TNFd and TNFa2 are associated with high levels of TNF-α while TNFa6 correlates with low levels of TNF-α [28]. Some microsatellite haplotypes have been associated with increased susceptibility to RA, specifically TNFa6;b5;c1;d3;e3 [29].

#### 2. IL-1

In addition to TNF, IL-1 is another cytokine considered to be a major contributor to chronic destructive arthritis. It is generally accepted that arthritis can be induced in mice by injection of recombinant cytokines (TNF or IL-1) into the knee joint [30]. The biological activity of IL-1 depends on the balance between two proinflammatory cytokines (IL-1α and IL-1β) and a related anti-inflammatory protein, the IL-1 receptor antagonist (IL-1RA). The latter protein blocks the binding of IL-1α and IL-1β to the receptor. IL-1 is a particularly potent cytokine because it induces the suppression of matrix synthesis by the chondrocytes and the release of active aggrecanase, the dominant enzyme responsible for proteoglycan loss [30].

The genes encoding these three proteins (IL-1α, IL-1β and IL-1RA) have been mapped to a 430 kb region on chromosome 2 [31]. SNPs and other variations have been identified in each of the genes, which, together with linkage disequilibrium across the region, lead to the presence of common haplotypes in the population [32]. Among polymorphisms of particular interest are (i) biallellic SNPs in the IL-1α gene at position -889 C/T [33] and in exon 5 at +4845 G/T [34], (ii) in the IL-1β gene at position -511 C/T [35] and in exon 5 at +3953 C/T [36], (iii) in the IL-1RA gene at position +2018 C/T in exon 2 [37], and a (iv) penta-allelic polymorphic site in intron 2 containing variable numbers of an 86-bp tandem repeat (VNTR) sequence. Studies have reported an association with the presence of the rarer alleles at IL-1α (+4845) or IL-1β (+3953) and an increased susceptibility to RA [38], as well as an increased severity of joint destruction [39]. Other studies have related IL-1α (-889), IL-1α (+4845), IL-1β (+3953) and IL-1RA VNTR allele 2 polymorphisms to altered IL-1 production [40-42]. It has also been suggested that the genotype at IL-1β may influence IL-1RA levels [40]. IL-1RA +2018 C/T gene polymorphism may also have a pro-inflammatory effect [43].

#### 3. IL-6

IL-6 is another pleiotropic cytokine with a wide range of biological activities, including regulation of immune response, inflammation, haematopoiesis and bone metabolism [44]. The constitutive overproduction of IL-6 is thought to play a pathogenic role in RA. Serum IL-6 levels are reported to correlate with disease activity and radiographic joint damage [45]. However, other findings suggest that IL-6 may act as an anti-inflammatory mediator [46]. IL-6 has been observed to augment circulating levels of IL-1RA and soluble TNF receptor, both of which could have important anti-inflammatory effects by suppressing the action of IL-1 and TNF [47].

IL-6 promoter polymorphisms have also been described. The G/C transversion at position -174 and the G/A transition at position -622 are the most analyzed. These two polymorphisms are in complete linkage disequilibrium [48]. The -174 position polymorphism has been shown to affect IL-6 levels and has been associated with systemic juvenile chronic arthritis [49], although more recent data appear to rule out any important role of these polymorphisms in susceptibility to RA [48,50]. 

#### 4. IL-10

IL-10 is another cytokine that mediates down-regulation of the inflammatory response. Specifically, IL-10 acts as a negative autocrine regulator of TNF-α and other proinflammatory cytokines [51]. Allelic polymorphisms in the gene for IL-10, which is located on chromosome 1, affect the respective levels of cytokine produced. Thus, allelic mutations at positions -1082 G to A, -819 T to C and -592 A to C may result in an ACC haplotype associated with lower expression levels of IL-10 [52]. These allelic variations are also correlated with autoimmune manifestations [53], in particular, the -1082AA genotype is associated with RA in women [54]. However, these IL-10 promoter polymorphisms were not important for development or severity of RA in Colombian population [55]. In contrast, the IL-10 -1082GG genotype is associated with an up-regulation of IL-10 production in lymphocytes [12].

## PHARMACOGENETICS OF ANTIRHEUMATIC DRUGS

### Disease Modifying Antirheumatic Drugs (DMARDs) in RA

This class of drugs potentially reduces or prevents joint damage and preserves joint integrity and function by affecting the immune system. However, the outcome of treatment with these drugs in RA patients is quite variable and unpredictable. The cause for differences in efficacy and occurrence of adverse drug reactions may be genetic variation in drug metabolism among individuals. 

Among the DMARDs that have the most potential for use in “tailor-made drug therapy” for RA patients are methotrexate, sulfasalazine and azathioprine.

#### 1. Methotrexate

Methotrexate (MTX) is one of the most widely used DMARDs for treatment of RA. The principal pharmacological effect of MTX is thought to be antagonism for folate. MTX enters the cell through reduced folate carrier-1 (RFC-1) and is intracellularly converted to MTX polyglutamates. Polyglutamation of MTX enhances the intracellular retention of MTX and promotes the inhibition of de novo purine synthesis along with the buildup of adenosine, a potent anti-inflammatory agent [56]. MTX directly inhibits several enzymes of the folate pathway, including dihydrofolate reductase (DHFR), thymidilate syntase (TYMS) and 5-amino-imidazole-4-carboxamide ribonucleotide (AICAR) transformylase (ATIC). Other folate enzymes are not directly inhibited by MTX, such as methylenetetrahydrofolate reductase (MTHFR), but their expression level may contribute to the antifolate effects of MTX [57]. Several polymorphisms in genes related to MTX transport across the cell membrane and the enzymes involved in the cellular metabolic pathway of MTX have been described (Table **2**).

Polymorphisms influencing MTX transport across the cell membrane include the reduced folate carrier 1 (RFC-1) G80A and adenosine triphospate-binding cassette B1 (ABCB1) C3435T. ABCB1 encodes a membrane transporter (P-glycoprotein) implicated in the disposition and bioavailability of several drugs. Genetic variations in these genes would affect the response to MTX in RA because both genes are involved in MTX transport. Individuals having the RFC 80A/A genotype have a greater response to MTX than patients with the wild-type allele (80G/G) [58]. Patients with the ABCB1 3435C/C and 3435C/T genotypes are at greater risk for active RA compared with patients with the 3435T/T genotype, who are better MTX responders [59].

Among the gene polymorphisms that influence metabolic enzymes in the cellular pathway of MTX, of particular note are two SNPs associated with the most studied gene in the MTX cellular pathway, that for methylenetetrahydrofolate reductase (MTHFR). This enzyme is very important in regeneration of reduced folate. The MTHFR C677T polymorphism results in a thermolabile variant of MTHFR with decreased enzyme activity [60]. A wide range of clinical effects is associated with this polymorphism, such as increased gastrointestinal side effects [61], increased hepatic toxicity [62] and overall adverse events [63]. A recent report shows that carriers of the MTHFR 677TT genotype are less likely to respond to MTX than those with alternate genotypes [64]; however, other authors have not found any effects on toxicity and efficacy [65, 66]. The A1298C polymorphism also results in decreased MTHFR activity and exhibits other discrepancies in its clinical effects. Some studies of carriers of this allele suggest increased MTX efficacy [62,63], increased susceptibility to RA [65] and increased risk of toxicity [64,67,68]; however, another study did not find any effects on efficacy and toxicity [66].

The genes for thymidylate synthase (TYMS) and aminoimidazole carboxamide ribonucleotide transformylase (ATIC) are also related to the cellular pathway of MTX. In fact, these enzymes are targets of MTX. TYMS is a key enzyme in de novo thymidylate synthesis, converting deoxyuridine monophosphate (dUMP) to deoxythymidine monophosphate (dTMP). This enzyme is inhibited by polyglutamated MTX. A polymorphic tandem repeat sequence has been identified in the 5´-untranslated region (5´-UTR) of the TYMS gene, with a variable number of 28-bp repeat elements [69]; the higher the number of repeat elements, the higher the mRNA expression and enzyme activity [70] and the less the efficacy of MTX [66]. Another polymorphism, consisting of a 6-bp deletion of the sequence TTAAAG at nucleotide 1494 in the 3´-UTR of TYMS, has been described [71]. This deletion may be associated with decreased TYMS mRNA stability and expression [72], thereby increasing MTX efficacy [66].

ATIC converts aminoimidazole carboxamide ribonucleotide (AICAR) to 10-formyl AICAR. ATIC is directly inhibited by MTX, leading to accumulation of both AICAR and adenosine, an anti-inflammatory purine. A previous study determined that homozygosity for the C347G polymorphism in ATIC, the double-repeat allele in TYMS or the RFC1 G80A SNP appeared to correlate with increased MTX response [73].

Polymorphisms have been described in other genes in the MTX pathway, such as γ-glutamyl hydrolase (GGH), DHFR or methionine synthase (MS), although their functional and clinical significance remain unknown.

#### 2. Sulfasalazine

Sulfasalazine (SASP) is another DMARD commonly used to treat RA. However, the use of SASP is limited by the occurrence of adverse effects in some individuals [74]. After oral intake, SASP is cleaved by intestinal bacteria into 5-amino salicyclic acid and sulfapyridine. Sulfapyridine is then metabolized in the liver by acetylation. The N-acetyltrans-ferase 2 (NAT2) gene, located on chromosome 8p22, encodes the enzyme involved in the acetylation of sulfapyridine and can be polymorphic. Polymorphisms in the NAT2 gene (Table **3**), which influence the slow *versus* fast acetylator status of individuals, are the direct determinant of acetylator status. Slow acetylators are more prone to toxicities from SASP compared to fast acetylators [75].

The wild-type NAT2*4 allele encodes the rapid acetylator status; though there are a large number of NAT2 variants, those for NAT2*5A, NAT2*5B, NAT2*5C, NAT2*6 and NAT2*7, which consist of several SNP combinations in exon 2 of the gene, are the most common variants that encode the slow acetylator status by means of decreased activity of the NAT2 enzyme. Because of the slow acetylation, these variants are associated with increased concentrations of toxic SASP intermediates [76,77].

The acetylator status of an individual, which appears to be influenced by NAT2 polymorphisms, seems to be important in determining the risk of toxicity from SASP. Thus, assays to prospectively identify the NAT2 genotype in patients before SASP administration will be powerful tools in clinical practice for averting SASP toxicity [78].

#### 3. Azathioprine

Azathioprine (AZA) is widely used to treat malignancies, rheumatic diseases and solid organ transplant rejection. AZA is now used less frequently for treatment of RA because of the changes in the treatment system along with the development of other DMARDs [77]. Thiopurine methyltransferase (TPMT) is one of major enzymes involved in the metabolism of AZA. Population studies have established that the activity of TPMT in erythrocytes is trimodal: approximately 90% of the population has high activity, 10% has intermediate activity and 0.3% has low or no activity [79]. 

Three allelic variants of the TPMT gene: TPMT*2 (G238C), TPMT*3A (G460A and A719G), TPMT*3C (A719G) (Table **4**) account for 60-95% of persons with low or intermediate TPMT activity [80]. Clinically, these polymorphisms are associated with hematological and gastrointestinal toxicity [81]. Thus, TPMT genotyping would be helpful for predicting AZA toxicity.

### Biological Agents in RA

The introduction of biological agents has dramatically improved the treatment of RA. These agents not only reduce the symptoms and signs of the disease, they also slow down the radiographic progression of disease [82]. However, these therapies are substantially more expensive than traditional DMARDs and are not effective in all patients [83]. In fact, there is consistent evidence that 25% to 30% of RA patients fail to respond [84]. The early identification of patients who will respond positively to these drugs would greatly help to establish a cost-effective treatment profile for the use of these molecules [85].

Intensive studies on the TNF and IL-1 driven inflammation processes have led to the development of cytokine blockers for RA treatment. Three TNF blockers are currently approved by the Food & Drug Administration (FDA) for the treatment of RA: Etanercept, Infliximab and Adalimumab. These blockers are derived from either a recombinant TNF receptor, TNFRSF1B in the case of Etanercept, or an anti-TNF-α monoclonal antibody for Infliximab and Adalimumab. The molecular mechanism of these TNF blockers, although quite different, involves inhibition of the binding of TNF to cell surface TNF receptors, thereby blocking signal transduction pathways induced or regulated by TNF. Although anti-TNF-α neutralizing therapy can be highly effective in reducing symptoms and signs of RA, not all patients show the same degree of response in disease progression [86]. It has been suggested that variability in the promoter and coding regions of the TNF-α gene may modulate the magnitude of the secretory response of this cytokine [87].

The fourth biological agent approved by the FDA for the treatment of RA is Anakinra, a recombinant form of IL-1RA whose molecular mechanism was explained above.

The drugs with the potential for use in “tailor-made drug therapy” for RA patients share problems relating to efficacy and toxicity. An increased risk of lymphoma is one example of the toxicity associated with these drugs. However, we must take into account the fact that patients with severe RA have approximately a two-fold increased risk of lymphoma. Some of this increased risk reflects an increase in Epstein-virus-associated lymphomas. This may be related to the elevated Epstein-Barr virus load found in RA patients and may reflect the subtle impairment of antiviral immunity in this group of patients [88].

#### 1. Etanercept

**Fig. (2):**
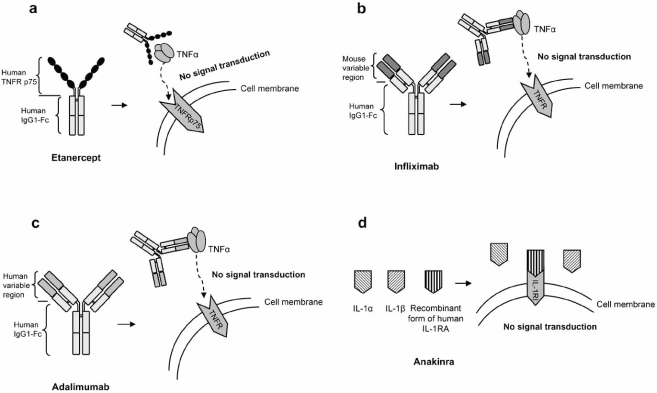
Molecular mechanism of biologic therapies: **A**) The tumor necrosis factor-α (TNF-α) blocker, Etanercept. **B**) The tumor necrosis factor-α (TNF-α) blocker, Infliximab. **C**) The tumor necrosis factor-α (TNF-α) blocker, Adalimumab. **D**) The interleukin-1 (IL-1) blocker, Anakinra, a recombinant form of the human IL-1 receptor antagonist (IL-1RA).

Etanercept is a dimeric fusion protein that joins the human p75 TNF receptor to the FC domain of human IgG1 (Fig. **2a**). This drug is made exclusively of human amino acidic sequences and has 934 amino acids.

The effectiveness of Etanercept for the treatment of RA, and early RA, has been proven both in monotherapy or combined with Methotrexate. The radiologically-assessed progression of joint degeneration decreases significantly when this drug is used for more than 24 months. Treatment with Etanercept alone shows significantly better clinical results than treatment with MTX alone.

Unfortunately, opportunistic infections, including tuberculosis, cardiac insufficiency and lymphoma, have been described in some patients treated with Etanercept. This risk increases if corticosteroids or other immunosupressive agents, such as MTX, are given concurrently. An association with demyelinating diseases has also been suggested [89].

#### 2. Infliximab

Infliximab is a monoclonal antibody that binds to and neutralizes the activity of TNF-α. It is a mouse/human chimera that joins the variable regions of a mouse antibody to the constant region of human IgG1 (Fig. **2b**). This drug was the first TNF blocker used in the treatment of RA and demonstrated the importance of TNF-α in the pathology of RA.

Important benefits of Infliximab include improved quality of life, prevention of structural articular damage and, possibly, bone repair. This drug has also been successfully used, both alone and combined with MTX, in treating other diseases such as Crohn´s, ankylosing spondylitis and psoriatic arthritis.

Regrettably, Infliximab shares some of the same problems as Etanercept, including infusion reactions, opportunistic infections, tuberculosis and increased risk of lymphoma [90]. Demyelinating processes, cardiac insufficiency and autoimmune disorders associated with Infliximab treatment have also been described.

#### 3. Adalimumab

Adalimumab is a fully human IgG1 monoclonal antibody whose molecular mechanism is similar to that of Infliximab. It binds to circulating and cell-surface TNF-α and blocks the interaction of TNF-α with the cell surface TNF receptors p55 and p75 (Fig. **2c**). Adalimumab also modulates the biological responses induced by TNF and reduces levels of IL-6 and MMPs (MMP-1 and MMP-3) [91].

Adalimumab is utilized either as a monotherapy or with MTX. An outstanding benefit of Adalimumab is that it inhibits the progression of the structural articular damage in long-term RA patients who have not experienced satisfactory response to DMARDs.

The toxic effects associated with the use of Adalimumab include the same problems seen with Etanercept and Infliximab: opportunistic infections, tuberculosis, demyelinating processes, autoimmune disorders and cardiac insufficiency.

#### 4. Anakinra

Anakinra is a recombinant form of human IL-1RA that acts as an antagonist to the biological activity of IL-1 by competitively inhibiting the binding of IL-1 to its cell membrane receptor, thus blocking cell signaling (Fig. **2d**). Its effectiveness in treating RA patients has been investigated either alone or combined with Etanercept, MTX, or other DMARDs [92,93,94]. Therapy with Anakinra in combination with Etanercept has not shown a clinical advantage and, at present, is contraindicated because of an increase in opportunistic infections.

The inhibition of the progression of structural damage in RA is an important benefit of Anakinra. However, an adverse reaction at the injection site is the major disadvantage of this drug. Pneumonias and cutaneous infections have also been described [94].

### Pharmacogenetics of Biological Agents

Several studies have correlated the response to these biological agents with some of the gene polymorphisms described in this review (Table **5**). Extended haplotypes spanning from HLA-DRB1 to the TNF region influence Etanercept response in Caucasian patients [95]. In the same study, the authors show that patients with two copies of the HLA-DRB1 shared epitope alleles had an improved response to Etanercept compared to patients without the allele or with one copy of the allele. Because of the large number of genes with roles in immune system function and regulation in the HLA region and the extensive linkage disequilibrium seen in this region, it is likely that a number of other genes influence treatment response [96].

In an extensive study, 78 patients treated with Infliximab were genotyped for HLA-DRB1, HLA-DQA1 and HLA-DQB1 alleles, a trinucleotide repeat polymorphism within the MHC class I chain-related gene A (MICA), and TNF microsatellites a through e, D6S273, BAT2 and D6S2223. The authors concluded that the D6S273_4/ BAT2_2 microsatellite haplotype pairs occurred significantly more often in responders than non-responders. Similarly, the frequency of the TNFa11;b4 haplotype, a marker usually found in context with D6S273_4/ BAT2_2, was increased and that of the D6S273_3 allele was decreased in responders [11]. These results led the authors to speculate that these markers occur on the same haplotype that carries an unknown “response gene.”

In other studies, a correlation between both the TNF-α -308G [12] and TNF-α -857T polymorphisms and good response to Etanercept has been shown [97]. Other authors have studied the response to Etanercept or Infliximab in patients with severe RA, characterized by non-responsiveness to MTX, in combination with other DMARDs. Those patients with the TNFRSFB 196TT genotype had a higher level of responsiveness to anti-TNF therapy over 24 weeks than did patients with the TG/TG genotype. Based on these results, the 196TT genotype correlates with a higher response to anti-TNF therapy in RA, while the presence of the G allele correlates with a reduced response [98].

The combination of TNF -308 G/G with IL-10 -1082G/G genotypes (patients with lower inflammatory response) also showed a better response to Etanercept. Thus, Etanercept appears to be more effective in patients carrying a genotype encoding for a lower inflammatory response [12]. Another study showed that IL-10 promoter microsatellite polymorphisms are associated with improved response to long term treatment with Etanercept [99].

Pharmacogenetic studies on the effectiveness of Infliximab have also been performed. The -308 G/A SNP in the TNF promoter region [100,101,102] was found to influence the response to Infliximab; those with the G/G genotype were better responders. Some authors speculate that the TNF -308 polymorphism influences the response to Infliximab through its effects on circulating TNF levels [99]. The presence of the A allele (high TNF production) would correlate with a poorer response to Infliximab. However, other studies of TNF -308 G/A polymorphism reported no association with response to Infliximab [98,11]. In the same way, a study carried out by our group in 113 RA subjects showed that TNF-α gene promoter polymorphisms, G-308A and G-238A, and the SE and DR3 alleles do not correlate with the response to Infliximab after 30 weeks quantified by improvement of DAS 28 [103].

To our knowledge, the only pharmacogenetic study of the responsiveness to Anakinra showed a highly significant association between the presence of the rarer T allele at IL-1α (+4845) and response to Anakinra [104]. 

## CONCLUSIONS

In summary, the response to therapy is probably partially determined by the genetic makeup of the individual. As stated previously, RA is a disease defined by well accepted criteria [105], but its clinical features and the molecular pathways involved are heterogeneous [106]. Therefore, the response to different treatments varies considerably among individual patients. With the development of a variety of costly new drugs and a lack of complete information on side effects, such as susceptibility to infection, the need for genetic markers prognostic for treatment response is increasing [107]. These genetic markers may lie in some of the genes described above, genes encoding the proteins involved in the drug target, drug metabolism or disease pathogenesis [108]. In this sense, the results of a study by Lequerré *et al*. are of particular interest [109]. They obtained a gene profiling of 41 mRNA transcripts suitable to predict the likely response to treatment with the combination of infliximab and MTX from peripheral blood mononuclear cells. An understanding of the genetic contribution to treatment success will become more and more relevant as therapies begin to target the key mechanisms of RA pathology [107].

To date, many polymorphisms in TNF, TNFRSF1B, MHC class alleles and other cytokine genes have been described; however their functional significance remains controversial. Some studies have produced conflicting results; population stratification and linkage disequilibrium have been cited as potential causes for the inability to replicate results of genetic association studies [110]. It is possible that analysis of haplotypes in candidate regions, rather than individual SNPs, may be a more productive approach. Because pharmacogenetics is a nascent field in which studies are just emerging, it is tempting to speculate that tailor-made cytokines or other specific molecule-directed treatments based on individual genotype will be applicable in the near future [30]. To achieve this individually-tailored approach to therapy, large prospective studies involving multiple institutions will be required to obtain adequate patient numbers to determine whether genetic variants of cytokines and other specific molecules contribute directly to either the pathophysiology of RA or the response to treatments.

## Figures and Tables

**Table 1:** Gene Polymorphisms in Rheumatoid Arthritis (RA)

Gene Symbol	Polymorphism Position	Alleles	Possible Effect of Polymorphism	References
*TNFα*	+1304	G A	May contribute to the susceptibility to RA / Possible linkage disequilibrium	[22]
	+489	G A	More severe erosive disease	[16]
	-238	G A	More severe articular erosions Less severe articular erosions	[20]
	-308	G A	Normal production of TNFα Up-regulation of TNFα production	[19, 18]
	-857	C T	May contribute to the susceptibility to RA / High TNFα production	[23]
	-863	C A	May contribute to the susceptibility to RA / High TNFα production	[21]
	-1031	T C	May contribute to the susceptibility to RA / High TNFα production	[22, 24]
	TNFa6;b5;c1;d3;e3		Increased susceptibility to RA	[29]
TNFRSF1B	codon 196	T G	More effective in increasing IL-6 production	[26]
*IL-1*	IL-1α -889	C T	Altered production of IL-1α	[42]
	IL-1α +4845 (exon 5)	G T	Altered production of IL-1α / Increased susceptibility to RA	[34, 38, 41]
	IL-1β -511	C T	Altered production of IL-1RA	[40]
	IL-1β +3953 (exon 5)	C T	Altered production of IL-1RA / Severe joint destruction	[40, 41, 39]
	IL-1RA +2018 (exon2)	C T	Possible pro-inflammatory effect	[43]
*IL-6*	-174	G C	Reduced production of IL-6	[49]
	-622	G A	Reduced production of IL-6	[48]
*IL-10*	-1082	G A	Up-regulation of IL-10 production in lymphocytes Lower expression levels of IL-10 / Associated with RA in women	[12] [52, 54]
	-819	T C	Lower expression levels of IL-10 / Autoimmune manifestations	[52, 53]
	-592	A C	Lower expression levels of IL-10 / Autoimmune manifestations	[52, 53]
HLA	Specific shared epitope alleles (HLA-DR)		May increase susceptibility to and severity of RA	[8-10]

TNF-α = tumor necrosis factor-α; TNFRSF 1B = TNFreceptor; IL-1 = interleukin-1; IL-6 = interleukin-6; IL-1RA = IL-1 receptor antagonist; HLA = Human Leukocyte Antigen.

**Table 2:** Pharmacogenetics of Methotrexate (MTX) in Rheumatoid Arthritis (RA)

Gene Symbol	Polymorphism	Effect of Polymorphism	Pharmacogenetics	References
RFC1	G80A	Increased MTX entry into cell	Increased response to MTX	[58]
ABCB1	C3435T	Involved in MTX transport	Increased response to MTX	[59]
MTHFR	C677T	Thermolabile variant of MTHFR enzyme with decreased enzyme activity	Increased gastrointestinal side effects and increased hepatic toxicity	[61, 62]
			Overall adverse events	[63]
			No effects on toxicity or efficacy	[65, 66]
	A1298C	Decreased MTHFR activity	Increased MTX efficacy	[62, 63]
			Increased susceptibility to RA	[65]
			No effects on toxicity or efficacy	[64, 66]
			Increased risk of toxicity	[64, 67, 68]
TYMS	5´UTR 28 bp repeat	Increased mRNA expression and enzyme activity	Decreased MTX efficacy	[66]
	3´UTR 6bp deletion	Decreased mRNA stability and expression	Increased MTX efficacy	[66]
ATIC	C347G	AICAR accumulation and increased adenosine	In combination with double-repeat allele in TYMS or the RFC1 G80A SNP seems to correlate with an increased response to MTX	[73]

RFC1 = reduced folate carrier 1; ABCB1 = adenosine triphospate-binding cassette B1; MTHFR = methylenetetrahydrofolate reductase; TYMS = thymidylate synthase; ATIC = aminoimidazole carboxamide ribonucleotide transformylase; SNP = single nucleotide pohymorphism.

**Table 3:** Pharmacogenetics of Sulfasalazine (SASP) in Rheumatoid Arthritis (RA)

Gene Symbol	Polymorphism	Effect of Polymorphism	Pharmacogenetics	References
NAT2	NAT2*4	Increased activity of NAT2 enzyme	Decreased concentrations of SASP intermediates	[75]
	(wildtype)	(fast acetylator status)	Less prone to toxicities from SASP	
	NAT2*5A	Decreased activity of NAT2 enzyme	Increased concentrations of SASP intermediates	[76, 77]
	T341C,C481T	(slow acetylator status)	More prone to toxicities from SASP	
	NAT2*5B	Decreased activity of NAT2 enzyme	Increased concentrations of SASP intermediates	[76, 77]
	T341C,C481T,A803G	(slow acetylator status)	More prone to toxicities from SASP	
	NAT2*5C	Decreased activity of NAT2 enzyme	Increased concentrations of SASP intermediates	[76, 77]
	T341C,A803G	(slow acetylator status)	More prone to toxicities from SASP	
	NAT2*6	Decreased activity of NAT2 enzyme	Increased concentrations of SASP intermediates	[76, 77]
	C282T,G590A	(slow acetylator status)	More prone to toxicities from SASP	
	NAT2*7	Decreased activity of NAT2 enzyme	Increased concentrations of SASP intermediates	[76, 77]
	C282T,G857A	(slow acetylator status)	More prone to toxicities from SASP	

NAT2 = N-acetyltransferase 2.

**Table 4:** Pharmacogenetics of Azathioprine (AZA) in Rheumatoid Arthritis (RA)

Gene Symbol	Polymorphism	Effect of Polymorphism	Pharmacogenetics	References
TPMT	TPMT*2	Low to intermediate activity of TPMT	Hematological and gastrointestinal toxicity	[81]
	G238C	Decreased methylation of AZA		
	TPMT*3A	Low to intermediate activity of TPMT	Hematological and gastrointestinal toxicity	[81]
	G460A, A719G	Decreased methylation of AZA		
	TPMT*3C	Low to intermediate activity of TPMT	Hematological and gastrointestinal toxicity	[81]
	A719G	Decreased methylation of AZA		

TPMT = thiopurine methyltransferase.

**Table 5:** Pharmacogenetics of Biological Agents in Rheumatoid Arthritis (RA)

Gene Symbol	Polymorphism Position	Alleles	Possible Effect of Polymorphism	Pharmacogenetics	References
*TNFα*	+489	G A	More severe erosive disease	No effect on response to Etanercept	[95]
	-238	G A	More severe articular erosions Less severe articular erosions	No effect on response to Etanercept	[95]
	-308	G A	Normal production of TNFα Up-regulation of TNFα production	Increased response to Infliximab	[100, 101]
	-857	C T	Susceptibility to RA / High TNFα production	Increased response to Etanercept	[97]
	TNFa11;b4	haplotype	Influence production of TNFα / Found with D6S273_4/BAT2_2	Increased response to Infliximab	[11]
TNFRSF1B	codon 196	T G	More effective in increasing IL-6 production	Increased response to anti-TNF therapy	[98]
*IL-1*	IL-1α +4845 (exon 5)	G T	Altered production of IL-1α / Increased susceptibility to RA	Increased response to Anakinra	[104]
*IL-10*	-1082	G A	Up-regulation of IL-10 production in lymphocytes Lower expression levels of IL-10 / Associated with RA in women	Increased response to Etanercept in combination with TNFα -308G/G	[12]
	-819	T C	Lower expression levels of IL-10 / Autoimmune manifestations	Patients with low inflammatory haplotype	
	-592	A C	Lower expression levels of IL-10 / Autoimmune manifestations	are better Etanercept responders	
HLA	Specific shared epitope alleles (HLA-DR)		May increase susceptibility to and severity of RA	Specific HLA-DRB1 alleles and haplotypes markers of better response to Etanercept	[95]
	HLA microsatelites BAT2,D6S273,D6S2223		Haplotype may carry "response gene"	D6S273_4/BAT2_2 haplotype correlates with increased response to Infliximab	[11]

TNF-α = tumor necrosis factor-α; TNFRSF1B = TNFreceptor; IL-1 = interleukin-1; IL-10 = interleukin-10.
